# Willingness of Taiwan’s Healthcare Workers and Outpatients to Vaccinate against COVID-19 during a Period without Community Outbreaks

**DOI:** 10.3390/vaccines9030246

**Published:** 2021-03-12

**Authors:** Shikha Kukreti, Mei-Yun Lu, Yi-Hsuan Lin, Carol Strong, Chung-Ying Lin, Nai-Ying Ko, Po-Lin Chen, Wen-Chien Ko

**Affiliations:** 1 Department of Public Health, National Cheng Kung University Hospital, College of Medicine, National Cheng Kung University, Tainan 701, Taiwan; t88087025@gs.ncku.edu.tw (S.K.); n967733@mail.hosp.ncku.edu.tw (M.-Y.L.); carolcj@ncku.edu.tw (C.S.); 2 Center for Infection Control, National Cheng Kung University Hospital, Tainan 701, Taiwan; 3 Department of Nursing, National Cheng Kung University Hospital, College of Medicine, National Cheng Kung University, Tainan 701, Taiwan; t26091018@gs.ncku.edu.tw (Y.-H.L.); nyko@mail.ncku.edu.tw (N.-Y.K.); 4 Institute of Allied Health Sciences, College of Medicine, National Cheng Kung University, Tainan 701, Taiwan; 5 Department of Occupational Therapy, College of Medicine, National Cheng Kung University, Tainan 701, Taiwan; 6 International Doctoral Program in Nursing, Department of Nursing, College of Medicine, National Cheng Kung University, Tainan 701, Taiwan; 7 Center for Infection Control, Department of Internal Medicine, National Cheng Kung University Hospital, College of Medicine, National Cheng Kung University, Tainan 701, Taiwan; cplin@mail.ncku.edu.tw (P.-L.C.); winston@mail.ncku.edu.tw (W.-C.K.)

**Keywords:** COVID-19, healthcare workers, outpatient, willingness, vaccination

## Abstract

To control the spread of the novel coronavirus disease 2019 (COVID-19), COVID-19 vaccination has been quickly developed. However, the COVID-19 pandemic will not be controlled if the COVID-19 vaccination uptake willingness is low. Therefore, the study aim was to explore the COVID-19 vaccination uptake willingness among the outpatient population and healthcare workers in Taiwan during the worldwide pandemic period without community outbreaks. A cross-sectional survey was conducted among healthcare workers (HCWs; *n* = 500; mean age = 32.96 years) of National Cheng Kung University Hospital (NCKUH) and outpatients (*n* = 238; mean age = 34.43 years) arriving at NCKUH. We used an online survey conducted between September 24 and 21 November 2020, for healthcare workers, and between 27 October and 31 December 2020, for the outpatient sample. Information regarding willingness to receive vaccination, willingness to rapid test, fear of COVID-19, risk perception, and preventive behaviors was collected in both samples; information regarding willingness to care for patients was collected in healthcare workers. Willingness to receive vaccination was the main variable in the present study; willingness to rapid test, willingness to care for patients, fear of COVID-19, risk perception, and preventive behaviors were the secondary variables in the study. The factors associated with vaccination willingness were identified through logistic regression analysis. The participants’ willingness to receive vaccination was low for both healthcare workers (23.4%) and the outpatient sample (30.7%). Similarly, their willingness to take rapid tests was low (23.6% for healthcare workers and 28.6% for outpatient sample). Risk perception (crude odds ratio (COR) = 1.29; 95% confidence interval (CI) = 1.03, 1.63), willingness to take rapid test (COR = 9.24; 95% CI = 5.76, 14.83), and preventive COVID-19 infection behaviors (COR = 2.32; 95% CI = 1.52, 3.56) were significant factors explaining the healthcare workers’ willingness to receive vaccination. Willingness to take a rapid test (COR = 8.91; 95% CI = 4.71, 16.87) and preventive COVID-19 infection behaviors (COR = 1.69; 95% CI = 1.09, 2.60) were significant factors explaining the outpatient sample’s willingness to receive vaccination. Willingness to vaccinate against COVID-19 among HCWs and outpatients is low due to the relatively safe status of COVID-19 infection in Taiwan. These findings can help policymakers advocate for the effectiveness of and provide transparent information on COVID-19 vaccination uptake in a country/region with a relatively safe COVID-19 outbreak status.

## 1. Introduction

The novel coronavirus disease 2019 (COVID-19) was discovered in late 2019 and resulted the worldwide pandemic [1]. The threat of COVID-19 transmission is ongoing, given several variants that have been identified [2]. The impacts of COVID-19 have far exceeded its high mortality rate [3]: psychological and social health have been largely impacted worldwide due to the direct and indirect effects of COVID-19 [4,5,6,7,8,9,10,11,12,13,14,15,16,17,18,19,20]. The direct impacts include psychological distress and mental health problems, given the fear of COVID-19 [4,5,6,7,8,9]. The indirect impacts may be attributed to the changes in human lifestyles, which have occurred due to some policies implemented to control the spread of COVID-19 infection [10,20,21]. These changed lifestyles (e.g., lack of outdoor activities due to lockdown) may also impair individuals’ mental health [10,11,13,14,15,16,19,22]. Therefore, the entire world is awaiting COVID-19 vaccination to effectively and efficiently control the transmission of COVID-19 infections.

However, prior evidence showed that individuals may be reluctant to receive vaccination due to several reasons, including the 3 Cs model: little confidence in vaccination, high complacency due to perceived low risk of infection, and low convenience of getting vaccinated [23], in addition to economic issues [24,25,26]. Although prior evidence has indicated the benefits of vaccination, especially from the perspective of social protection and the effectiveness in preventing deaths (preventing 2 to 3 million deaths per year) [26], some studies have still reported low levels of willingness to receive COVID-19 vaccination [24,27,28], posing a global challenge for healthcare providers and policy makers to increase and promote COVID-19 vaccination coverage. Without a certain level of COVID-19 vaccination coverage, controlling COVID-19 infections will be difficult. Moreover, as the development of COVID-19 vaccination was accelerated (i.e., over 160 candidate vaccines have been tested with around 20 being under clinical evaluation worldwide) [29,30,31], healthcare providers and policy makers may need to advocate and promote COVID-19 vaccination to achieve a high level of vaccination coverage. Thus, the social protection can be maximized, and the threat of COVID-19 can be minimized.

The issue of COVID-19 vaccination has thus been widely discussed in the current literature in different countries and regions [24,25,26,27,28,30,32,33,34,35,36,37,38,39,40,41,42,43,44,45,46,47,48,49,50]. From a literature review, we found that most published studies focused on the COVID-19 vaccination willingness in general populations. More specifically, a relatively high rate of COVID-19 vaccination uptake willingness (49.7% to 91.3%) has been observed in the general populations in some Asian countries, including mainland China [35], Malaysia [37], and Indonesia [32]; some Europe countries, including Turkey, France, Italy, the UK, Ireland, Denmark, Germany, Portugal, and the Netherlands [33]; and the U.S. [34,41,50]. Low levels of willingness toward COVID-19 vaccination (27.0% to 29.4%) have been documented in general populations in Nigeria [28] and Arab countries, including Jordan, Kuwait, and Saudi Arabia [27]. Aside from general populations, willingness toward COVID-19 vaccination has been investigated in other populations, including healthcare workers in the Republic of Congo (27.7% were willing to receive vaccination) [24], Hong Kong (40.0% to 63.0% were willing to receive vaccination) [25,46], and the Republic of Malta (61.8% were willing to receive vaccination) [42]. Of medical students in the U.S., 53.0% were willing to receive vaccination [40]. Of parents or guardians in the U.K., 90.1% were willing to receive vaccination [36], and 72.6% in mainland China [49]. Of caregivers in the U.S., Israel, Japan, Spain, and Switzerland, overall, 65.0% were willing to receive vaccination [43].

To the best of our knowledge, no studies have explored COVID-19 vaccination uptake willingness among outpatient populations, and only few studies have investigated the COVID-19 vaccination uptake among healthcare workers [24,25,42,46]. In addition, most of the evidence has been derived from countries/regions with serious COVID-19 outbreaks or those with serious COVID-19 community transmission [25,32,47,50]. To fill this literature gap, we investigated the COVID-19 vaccination willingness in two Taiwanese populations, healthcare workers and outpatients, during the pandemic period but without community transmission in the country. More specifically, the COVID-19 cases were well-controlled by the Taiwan government, with most of the confirmed cases being imported, and COVID-19-related deaths limited to under 10 people [51]. In addition, we explored potential factors that could explain the COVID-19 vaccination uptake willingness in the two samples, separately. 

## 2. Materials and Methods

### 2.1. Participants and Recruitment Procedure

We adopted a cross-sectional design with convenience sampling. There were two target samples in this study: the National Cheng Kung University Hospital (NCKUH) healthcare workers and the NCKUH outpatients. The NCKUH, with more than 5000 employees and more than 1500 beds, is the largest medical center in Southern Taiwan. We used a cross-sectional design to recruit 500 healthcare workers and 238 NCKUH outpatients who were willing to participate in this study. All the participants were approached using an online survey. Specifically, we sent out the survey link to the NCKUH healthcare workers and outpatients who had provided their email information in the NCKUH IT system. In the link, the study purpose and information (including the right to withdrawal) were clearly mentioned in the first page of the online survey. Only when the participant clicked on the button to agree to participate could they continue the online survey. The inclusion criteria for healthcare workers were (1) a healthcare worker in the NCKUH; (2) aged over 20 years; and (3) willing to participate in the study. The inclusion criteria for outpatients were (1) being a NCKUH outpatient; (2) aged over 20 years; and (3) willing to participate in the study. The survey period for healthcare workers was between 24 September and 21 November 2020; the survey period for the outpatient sample was between 27 October and 31 December 2020. We obtained Institute Review Board (IRB) approval (IRB number A-ER-109-149) from the NCKUH for this study prior to commencement.

The needed sample size for the present study was calculated as follows: For healthcare workers, we referenced a similar study (i.e., a healthcare worker population from Hong Kong [46]) to assume a prevalence rate of 40%, with 5% precision and a population size of 5200 (i.e., about the size of the NCKUH healthcare staff). We calculated an estimated sample size of 345. For outpatients, we referenced a similar study (i.e., a general population from mainland China [35]) to assume a prevalence rate at 83.3%, with 5% precision and a population size of 40,000 (i.e., about the number of NCKUH outpatients in two months). We estimated the sample size as 213. Therefore, we obtained sufficient responses from both healthcare workers (i.e., 500) and outpatients (i.e., 238) to investigate their COVID-19 vaccination uptake willingness.

### 2.2. Measures

#### 2.2.1. Background Information Sheet

Participants’ characteristics, including age (fill in a number with the unit of year), sex (male or female), and educational level (elementary school or below, junior high, senior high, bachelor’s degree, master’s degree, or Ph.D. degree) were self-reported by the participants. 

#### 2.2.2. Willingness to Receive Vaccination 

The item, “To what extent you would like to receive vaccination if not considering the price?” with a five-point Likert scale (1 = very unlikely, 2 = unlikely, 3 = neutral, 4 = likely, and 5 = very likely), was used to examine the willingness to receive vaccination. We then converted the item into a dichotomous scale, where original scores 1 to 3 were recoded as low willingness to receive vaccination and original scores 4 and 5 as high willingness. 

#### 2.2.3. Willingness to Take Rapid Test

The item, “To what extent you would like to take a rapid COVID-19 infection test if not considering price?” with a five-point Likert scale (1 = very unlikely, 2 = unlikely, 3 = neutral, 4 = likely, and 5 = very likely), was used to examine the willingness to take a rapid test. We then converted the item into a dichotomous scale, where original scores 1 to 3 were recoded as low willingness and original scores 4 and 5 as high willingness to take a rapid test. 

#### 2.2.4. Risk Perception

The item, “What is the likelihood that you may be infected by COVID-19?” with a five-point Likert scale (1 = very unlikely, 2 = unlikely, 3 = neutral, 4 = likely, and 5 = very likely), was used to examine the risk perception of the participants. 

#### 2.2.5. Fear of COVID-19

The Fear of COVID-19 Scale (FCV-19S) was used to assess fear of COVID-19. The FCV-19S consists of 7 items with a five-point Likert scale (1 = strongly disagree, 2 = disagree, 3 = neither disagree nor agree, 4 = agree, and 5 = strongly agree). The average score of the 7 items was then computed to obtain the level of fear for a participant. A higher score indicates greater fear, and the psychometric properties of the FCV-19S were found to be satisfactory [8]. 

#### 2.2.6. Preventive COVID-19 Infection Behaviors

Sixteen behaviors related to prevention of being infected by COVID-19 were used to examine the participants’ preventive COVID-19 infection behaviors. The 16 behaviors included those recommended by the World Health Organization (e.g., clean hands thoroughly, use elbow and handkerchief to cover mouth and nose when sneezing) and those listed in the validated Preventive COVID-19 Infection Behaviors Scale [8]. 

#### 2.2.7. Willingness to Care for Patients 

The item, “To what extent you would like to provide healthcare services to patients?” with a 0–10 visual analogue scale, was assessed for healthcare workers, only, and not for the outpatient sample. All the other measures were assessed for both healthcare workers and the outpatient sample. 

### 2.3. Statistical Analysis 

Descriptive statistics were first used to examine the participants’ characteristics, including their demographic information and measure scores. Then, univariate logistic regression models were used to examine the crude odds ratios (CORs) regarding how different variables (age, sex, fear of COVID-19, risk perception, willingness to take rapid test, preventive COVID-19 infection behaviors, and willingness to care for patients) explained the participants’ willingness to receive vaccination. Afterward, multivariate logistic regression models were used to examine the adjusted odds ratios (AORs) when the variables were simultaneously entered in the logistic regression models. All the CORs and AORs are presented with a 95% confidence interval (CI). Data retrieved from healthcare workers and the outpatient sample were separated in all the analyses. Willingness to care for patients was only analyzed for healthcare workers. All the statistical analyses were performed using the IBM SPSS 21.0 (IBM Corp., Armonk, NY, USA).

## 3. Results

The two samples were relatively young. For the sample of healthcare workers, the mean (SD) age was 32.96 (7.99) years; in comparison, the mean age of healthcare workers in the population was 34.8 years. The mean (SD) age was 34.43 (10.02) years for the outpatient sample; in comparison, the mean age of the outpatients in the population was 41.50 years. The proportion of male healthcare workers in the sample was small (40, 8.0%). For comparison, the ratio of men in healthcare workers in the population was 18.0%. For the outpatient sample, there were 79 male respondents (33.2%); in the population, this proportion is 48.8%. The educational level of the present samples was high: more than 80% of the participants had completed higher education. The participants’ willingness to receive vaccination was low (*n* = 117, 23.4%, for healthcare workers; *n* = 73, 30.7%, for the outpatient sample); similarly, their willingness to take a rapid test was low (*n* = 118, 23.6% for healthcare workers; *n* = 68, 28.6%, for outpatients). Table 1 lists participants’ scores on the survey measures

The logistic regression models showed that risk perception (COR = 1.29; 95% CI = 1.03, 1.63), willingness to take a rapid test (COR = 9.24; 95% CI = 5.76, 14.83), and preventive COVID-19 infection behaviors (COR = 2.32; 95% CI = 1.52, 3.56) were the significant factors explaining the healthcare workers’ willingness to receive vaccination. However, only willingness to take a rapid test (AOR = 9.06; 95% CI = 5.49, 14.94) and preventive COVID-19 infection behaviors (AOR = 1.89; 95% CI = 1.15, 3.10) remained significant in the multivariate logistic model (Table 2). Willingness to take a rapid test (COR = 8.91; 95% CI = 4.71, 16.87) and preventive COVID-19 infection behaviors (COR = 1.69; 95% CI = 1.09, 2.60) were significant factors explaining the outpatient sample’s willingness to receive vaccination. However, only willingness to take a rapid test (AOR = 8.24; 95% CI = 4.24, 16.02) remained significant in the multivariate logistic model (Table 2).

## 4. Discussion

**Figure 1:**
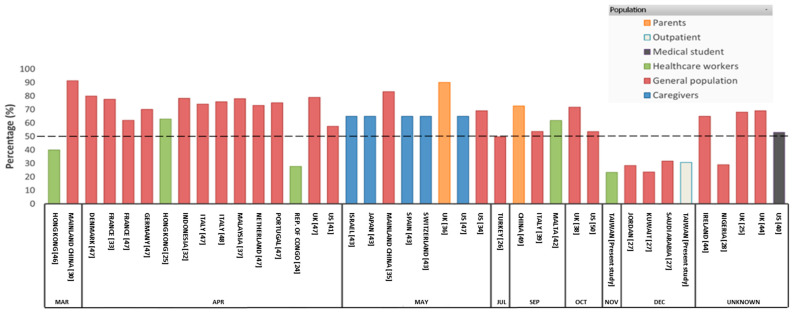
Willingness to receive COVID-19 vaccination as a percentage. The dashed line indicates 50% willingness to receive vaccination.

Through an online survey, with a cross-sectional design, we investigated the willingness toward COVID-19 vaccination uptake in two populations (healthcare workers and outpatients), which has been rarely discussed in the contemporary literature. Both samples reported a low level of willingness to receive COVID-19 vaccination, which may be due to the relatively safe status of COVID-19 infections in Taiwan. More specifically, during the survey period (24 September to 31 December 2020), the COVID-19 cases were controlled to under 800 with most (~88%) of the confirmed cases being imported [51]. Therefore, we postulate that individuals residing in Taiwan may not appreciate the importance of vaccination uptake. This postulation is partially based on the 3 Cs model (confidence, complacency, and convenience) [23]; that is, Taiwan residents might be aware of the low risk of being infected with COVID-19 and, thus, had high levels of complacency. However, we did not collect information regarding whether the COVID-19 infection status was the underlying reason for the low levels of willingness to receive COVID-19 vaccination. Therefore, we summarized the published evidence of the COVID-19 vaccination uptake willingness. Illustrated in Figure 1, high levels of willingness of COVID-19 vaccination uptake can be observed in most countries/regions. Low levels of willingness toward COVID-19 vaccination uptake were observed among healthcare workers in the Republic of the Congo (27.7%), general population in Nigeria (29.0%) [28], and the Arab countries (29.4%) [27], to which we can add our healthcare worker (23.4%) and outpatient (30.7%) samples. 

A review of the confirmed COVID-19 cases and deaths in the studies [24,25,26,27,28,30,32,33,34,35,36,37,38,39,40,41,42,43,44,45,46,47,48,49,50] that published information on COVID-19 vaccination uptake willingness somewhat supports our aforementioned postulation. The Republic of the Congo, which had low levels of willingness to receive COVID-19 vaccination in the general population (27.7%) [24], had relatively few confirmed COVID-19 cases (*n* = 220) and deaths (*n* = 9). All the other countries/regions with high numbers of COVID-19 cases and deaths reported high levels of COVID-19 vaccination uptake willingness (Table 3), except for the Arab countries [27]. The low levels of COVID-19 vaccination uptake willingness among Arab countries, where the burden of COVID-19 was severe, may be due to the low trust in the government and low levels of education [27]. Additionally, an increased level of willingness has been observed among Hong Kong healthcare workers, ranging from 40.0% to 63.0%, when an outbreak occurred [1,25,46]. This finding also indirectly supports the postulation mentioned above (i.e., the low levels of willingness found in the present study were due to the relatively low status in Taiwan). 

For both samples, the willingness to take a rapid test was the only factor that significantly explained the COVID-19 vaccination uptake willingness in the multivariate logistic regression models. Individuals with higher levels of willingness to take a rapid test may have higher levels of intent to avoid being infected by COVID-19. This finding echoes prior evidence in the relationship between willingness to get influenza vaccinated and diagnostic testing [52]. Therefore, willingness to take a rapid test is a good predictor of COVID-19 vaccination uptake willingness. Preventive COVID-19 infection behaviors were found to be significant factors in both samples in the univariate logistic regression models. This explains that the samples with higher compliance with preventive COVID-19 infection behaviors were likely to accept COVID-19 vaccination if not considering their willingness to take a rapid test. The significant association between preventive COVID-19 infection behaviors and COVID-19 vaccination uptake willingness was lower in outpatients and remained significant in healthcare workers when accounting for willingness to take a rapid test. This implies that the willingness to take rapid test and preventive COVID-19 infection behaviors may share the same underlying reasons explaining COVID-19 vaccination uptake willingness, that is, higher levels of intention to avoid being infected by COVID-19. 

There are some limitations to the present study. First, the study samples were recruited using convenience sampling, and the participants were affiliated with the NCKUH (either the hospital staff or hospital outpatients). Therefore, the representativeness of the samples is relatively low, and the generalizability of our findings is somewhat restricted. Following this limitation, the readers should be cautious with the high educational levels and young age of the study’s respondents. Future studies recruiting individuals with low educational levels and older age are thus needed. Second, we adopted a cross-sectional design; thus, causal relationships cannot be drawn. Therefore, future studies using longitudinal designs are warranted to further corroborate the significant association between the rapid test willingness and COVID-19 vaccination uptake willingness found in the present study. Third, some of the variables (e.g., rapid test willingness and COVID-19 vaccination uptake willingness) were not assessed using standardized instruments. Therefore, it is unclear whether there are any measurement biases in assessing these variables. 

## 5. Conclusions

Our findings demonstrated a low level of willingness to receive vaccination among Taiwanese individuals (23.4% of healthcare workers and 30.7% of outpatients). The low willingness may be due to the relatively safe status of COVID-19 in Taiwan, as evidenced by the high levels of willingness to receive vaccination among individuals residing in regions with a high risk of COVID-19 infection. Although several potential factors were found to significantly explain the COVID-19 vaccination uptake willingness, willingness to take a rapid COVID-19 test was the only significant factor in both samples in the controlled logistic regression models. According to the present findings, policy makers may want to advocate for the effectiveness of and provide transparent information on COVID-19 vaccination uptake in countries and regions that are relatively safe from COVID-19 outbreaks. COVID-19 vaccination uptake may start from those who have taken a rapid COVID-19 infection test to achieve initial vaccination coverage.

## Figures and Tables

**Table 1:** Participant characteristics.

Variable	Healthcare Workers (*N* = 500)	General Population (*N* = 238)
Age (years); Mean (SD)	32.96 (7.99)	34.43 (10.02)
Sex (Male); *n* (%)	40 (8.0)	79 (33.2)
Educational level (Senior high and below); *n* (%)	17 (3.4)	41 (17.2)
Fear of COVID-19; Mean (SD) ^a^	2.57 (0.82)	2.56 (0.96)
Risk perception; Mean (SD) ^a^	2.73 (0.93)	2.21 (0.87)
Willingness to take rapid test; *n* (%)	118 (23.6)	68 (28.6)
Willingness to receive vaccination; *n* (%)	117 (23.4)	73 (30.7)
Preventive behaviors; Mean (SD) ^a^	3.22 (0.51)	3.05 (0.68)
Willingness to care patients; Mean (SD) ^b^	6.07 (2.59)	--

^a^ Using 5-point Likert-type scale. ^b^ Using 0–10 Visual Analogue Scale.

**Table 2:** Logistic model examining the predictors on willingness to receive vaccination.

Independent Variable	COR (95% CI)	AOR (95% CI)
**Healthcare workers (*N* = 500)**		
Age	0.99 (0.96, 1.02)	0.98 (0.95, 1.01)
Gender (Ref: female)	1.64 (0.82, 3.29)	**2.53 (1.14, 5.65)**
Fear of COVID-19	1.10 (0.85, 1.41)	1.04 (0.76, 1.41)
Risk perception	**1.29 (1.03, 1.63)**	1.16 (0.89, 1.52)
Willingness to take rapid test (Ref: No)	**9.24 (5.76, 14.83)**	**9.06 (5.49, 14.94)**
Preventive behaviors	**2.32 (1.52, 3.56)**	**1.89 (1.15, 3.10)**
Willingness to care patients	1.08 (0.996, 1.17)	1.04 (0.94, 1.15)
**Outpatient population (*N* = 238)**		
Age	0.99 (0.96, 1.01)	0.99 (0.96, 1.02)
Gender (Ref: male)	1.17 (0.66, 2.09)	1.15 (0.58, 2.27)
Fear of COVID-19	1.03 (0.99, 1.07)	0.99 (0.94, 1.04)
Risk perception	1.35 (0.98, 1.87)	1.22 (0.84, 1.78)
Willingness to take rapid test (Ref: No)	**8.91 (4.71, 16.87)**	**8.24 (4.24, 16.02)**
Preventive behaviors	**1.69 (1.09, 2.60)**	1.27 (0.76, 2.12)

COR, crude odds ratio; AOR, adjusted odds ratio; CI = confidence interval. Significant odds ratios at *p* < 0.05 are presented in bold.

**Table 3:** Study review showing percentage of COVID-19 vaccination willingness and total cases, deaths, and population during the survey period.

Country/Region (Author, Year)	Population	Cases	Deaths	Population	Willingness (%)	Survey Period (2020)
Hong Kong [46]	Healthcare workers	715	4	7.45 million	40.00	26 February to March31
Mainland China [30]	General population	80,026	2912	1.39 billion	91.30	1 February to March30
Republic of Congo [24]	Healthcare workers	220	9	5.5 million	27.70	1 March to 30 April
Hong Kong [25]	Healthcare workers	1038	4	7.45 million	63.00	15 March to 30 April
Indonesia [32]	General population	2092	191	273.5 million	78.30	25 March to 6 April
France [33]	General population	114,657	20,246	65.2 million	77.60	26 March to 20 April
Italy [48]	General population	110,559	13,195	60.3 million	75.80	1 April
U.S. [41]	General population	818,510	43,685	331.0 million	57.60	16 April to 20 April
Denmark [47]	General population	6681	309	5.8 million	80.00	2 April to 15 April
France [47]	General population	106,206	17,152	67.0 million	62.00	2 April to 15 April
Germany [47]	General population	134,753	3804	83.0 million	70.00	2 April to 15 April
Netherland [47]	General population	28,153	3134	17.28 million	73.00	2 April to 15 April
Portugal [47]	General population	20,593	599	10.2 million	75.00	2 April to 15 April
U.K. [47]	General population	89,217	14,903	67.8 million	79.00	2 April to 15 April
Italy [47]	General population	162,479	21,131	60.3 million	74.00	2 April to 15 April
Malaysia [37]	General population	4683	76	32.3 million	78.00	3 April to 12 April
U.S. [43]	Caregivers	1,877,168	109,099	331.0 million	65.00	26 March to 31 May
Israel [43]	Caregivers	17071	285	9.05 million	65.00	26 March to 31 May
Japan [43]	Caregivers	16,851	891	126.3 million	65.00	26 March to 31 May
Spain [43]	Caregivers	251,913	29,050	46.9 million	65.00	26 March to 31 May
Switzerland [43]	Caregivers	30,862	1831	8.5 million	65.00	26 March to 31 May
U.K. [36]	Parents	202,085	31,571	67.8 million	90.10	19 April to 11 May
U.S. [34]	General population	1,877,168	109,099	331.0 million	69.00	1 May to 31 May
Mainland China [35]	General population	82,960	4634	1.39 billion	83.30	1 May to 19 May
Turkey [26]	General population	470,666	5323	84.3 million	49.70	10 June to 10 July
China [49]	Parents	85,134	4634	1.39 billion	72.60	1 September to 7 September
Italy [39]	General population	311,363	35,851	60.4 million	53.70	16 September to 28 September
Malta [42]	Healthcare workers	3035	34	4.42 million	61.80	25 September to 29 September
U.S. [50]	General population	7,854,367	217,136	331.0 million	53.60	June-1 to October7
U.K. [38]	General population	705,427	43,579	67.8 million	71.70	24 September to 17 October
Taiwan (The present study)	Healthcare workers	611	7	23.5 million	23.40	24 September to 21 November
Taiwan (The present study)	Outpatient	799	7	23.5 million	30.70	27 October to 31 December
Saudi Arabia [27]	General population	360,690	6101	34.27 million	31.80	14 December to 18 December
Jordan [27]	General population	271,514	3518	10.1 million	28.40	14 December to 18 December
Kuwait [27]	General population	147,531	916	4.20 million	23.60	14 December to 18 December
Nigeria [28]	General population	-			29.00	Unknown
U.S. [40]	Medical students	-			53.00	Unknown
U.K. [44]	General population	-			69.00	Unknown
Ireland [44]	General population	-			65.00	Unknown
U.K. [45]	General population	-			68.00	Unknown

## Data Availability

The data will be available upon reasonable request to the corresponding authors.
